# Subject-level differences in reported locations of cutaneous tactile and nociceptive stimuli

**DOI:** 10.3389/fnhum.2012.00325

**Published:** 2012-11-30

**Authors:** Peter Steenbergen, Jan R. Buitenweg, Jörg Trojan, Bart Klaassen, Peter H. Veltink

**Affiliations:** ^1^Biomedical Signals and Systems, Mira Institute for Biomedical Technology and Technical Medicine, University of TwenteEnschede, Netherlands; ^2^Department of Cognitive and Clinical Neuroscience, Central Institute of Mental Health, Medical Faculty Mannheim, Heidelberg UniversityMannheim, Germany

**Keywords:** perceptual map, touch, nociception, electrocutaneous stimulation, localization, body representations, primary representations

## Abstract

Recent theoretical advances on the topic of body representations have raised the question whether spatial perception of touch and nociception involve the same representations. Various authors have established that subjective localizations of touch and nociception are displaced in a systematic manner. The relation between veridical stimulus locations and localizations can be described in the form of a perceptual map; these maps differ between subjects. Recently, evidence was found for a common set of body representations to underlie spatial perception of touch and slow and fast pain, which receive information from modality specific primary representations. There are neurophysiological clues that the various cutaneous senses may not share the same primary representation. If this is the case, then differences in primary representations between touch and nociception may cause subject-dependent differences in perceptual maps of these modalities. We studied localization of tactile and nociceptive sensations on the forearm using electrocutaneous stimulation. The perceptual maps of these modalities differed at the group level. When assessed for individual subjects, the differences localization varied in nature between subjects. The agreement of perceptual maps of the two modalities was moderate. These findings are consistent with a common internal body representation underlying spatial perception of touch and nociception. The subject level differences suggest that in addition to these representations other aspects, possibly differences in primary representation and/or the influence of stimulus parameters, lead to differences in perceptual maps in individuals.

## Introduction

A number of reviews recently discussed the involvement of multimodal body representations in spatial perception of touch (Longo et al., 2010; Medina and Coslett, 2010; Serino and Haggard, 2010). Information from primary sensory representations with different reference frames is projected to these body representations, which allows, for instance, localizing cutaneous stimuli in space and integrating cues from different senses which are related to the body. Central processing of nociception and touch differs and these modalities may have different primary representations (Mancini et al., 2011). If this is indeed the case, there may be differences in spatial perception between these modalities.

For identifying the location of a stimulus on the body surface, somatosensory information needs to be referenced to models of body form and body surface (Longo et al., 2010; Medina and Coslett, 2010). In order to perform a pointing movement task to report the perceived location of the stimulus, this information needs to be translated into an external reference frame. This involves representations of body posture, which contain information about the position of body parts in space. There is evidence that these body representations are not perfectly matched; humans have been shown to exhibit systematic distortions in identifying the locations of landmarks in their hand, which indicates that representations of body shape differ from the physical shape of the body (Longo and Haggard, 2010, 2012). Furthermore, the relation between the reference frames of body form and body posture representations is variable, since the location of body parts in space is variable, while the shape of the body is constant. There is evidence that sensory information about the orientation of the head on the body and the eyes in the head are involved in aligning these representations (Pritchett and Harris, 2011; Pritchett et al., 2012). This alignment is not perfect, as is illustrated by the finding that tactile stimuli are mislocalized in the direction of gaze (Harrar and Harris, 2009).

Although much information is available on the cortical primary sensory structure of touch, the cortical representation of nociception is still a matter of debate. It has been suggested that SI is responsible for spatial perception of nociception as well as touch (Bushnell et al., 1999; Ogino et al., 2005). Activation patterns in SI differ for these modalities, with mechanical stimuli mainly activating areas 3b, 1 and 2, and heat pain stimuli additionally involving area 3a (Tommerdahl et al., 1998; Chen et al., 2009, 2011). Furthermore, touch and nociception may not lead to activity in the same cortical columns, analogous to the differences in activation between different tactile submodalities (Mountcastle, 1997; Friedman et al., 2004). Several researchers have argued that the somatotopy of cortical maps is fundamental to their functioning [see for instance Kaas (1997)], which is supported by a recent finding that experimental manipulations of the cortical topography of fingers affects reaction times in a decision task involving spatial perception (Wilimzig et al., 2012). Therefore, if SI is indeed involved in spatial perception of both touch and nocicepion, differences in cortical representation of these modalities may lead to differences in spatial perception. Alternatively, it has been suggested that the primary sensory cortex for nociception is located in the posterior insular-opercular region (Garcia-Larrea, 2012). Regardless of whether the primary sensory cortex for touch and nociception is the same or different, it is likely that the cortical representations for these modalities differ, which may lead to differences in spatial perception of these modalities. This supports the idea put forward by Mancini et al. (2011) that these modalities may have their own primary representations, which are mapped to the same multimodal internal body representations.

When humans localize a cutaneous stimulus, the reports generally deviate from the veridical stimulus site. When repeatedly stimulating various sites of a body part and asking a subject to localize these stimuli, a somatosensory perceptual map can be constructed which relates the localizations to the veridical stimulus sites (Trojan et al., 2006). Studies using this procedure in combination with mechanical and laser stimulation on the lower arm have shown that somatosensory perceptual maps deviate from the veridical stimulus locations for both touch (Trojan et al., 2010) and nociception (Trojan et al., 2006). The maps varied between participants: compared to the veridical stimulus locations, subjects exhibited overall biases and scaling of the area over which they reported. In a recent study we showed that somatosensory perceptual maps of non-painful electrocutaneous stimuli have highly reproducible features, which supports the idea that these maps measure a stable property of spatial perception and may therefore reflect internal body representations (Steenbergen et al., 2012b). Mancini et al. (2011) addressed the question whether the same body representations underlie spatial perception of the various cutaneous sensory modalities by comparing perceptual maps of tactile, heat and nociceptive stimuli on the hand. They found some significant differences in perceptual maps between stimulus modalities on the group level, but perceptual maps of the three modalities had similar features, from which the authors concluded that common internal body representations are involved in spatial perception of the modalities studied.

Multimodal body representations and primary representations in SI reflect each individual's own body. Differences between tactile and nociceptive SI representation may therefore vary between subjects. As a consequence, a resulting difference in somatosensory perceptual maps would also be subject-dependent and these individual differences therefore do not necessarily contribute to a difference at the group level. Therefore, we conducted a study in which we assessed agreement of tactile and nociceptive perceptual maps at the group level, as well as their differences at the subject level.

We conducted a study in which we compared perceptual maps of tactile and nociceptive electrocutaneous stimuli on the lower arm. Using stimulation electrodes designed for this study (Steenbergen et al., 2012a), we applied nociceptive and tactile stimuli at four sites. Based on the results by Mancini et al. (2011), we expected to find a small difference at the group level, but also to find some level of agreement between perceptual maps of the two modalities. Two topics which we were interested in were not addressed by Mancini et al. (2011). The first one was that we wanted to quantify the agreement between perceptual maps of the different modalities. We assessed this by calculating Intraclass Correlation Coefficients of the regression parameters fitted to the data of individual subjects. The second topic concerned differences between tactile and nociceptive localization in individuals rather than on the group level. We had no clear hypothesis on what type of differences to expect. We therefore tested for these differences in a way which required minimal assumptions about the data by conducting separate tests for all electrode sites and subjects.

## Materials and methods

### Subjects

Eighteen subjects from the population of students and employees of the University of Twente volunteered to participate in this study and gave informed consent prior to the experiments. One subject was excluded because he did not detect any of the nociceptive stimuli. The mean (M) age of the remaining seventeen subjects was 23.8 years with standard deviation (SD) of 2.7 years (range 19–28 years). Seven subjects were female. The arm length of the subjects was 27.2 ± 1.63 (M ± SD) cm, with the shortest being 24 cm and the longest 29 cm. The protocol was approved by the Medical Ethical Board Twente (file number NL35875.044.11).

### Stimulation method

**Figure 1:**
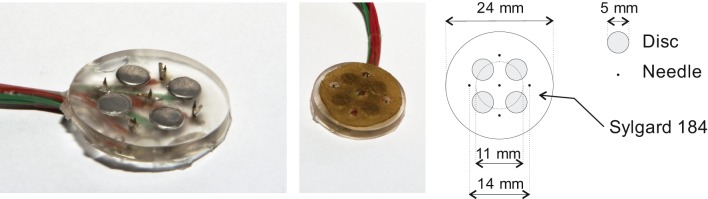
**One of the compound electrode arrays which were used in the experiments.** Each compound electrode consists of four disc electrodes and five needle electrodes. During experiments, the disc electrodes were covered with a conducting pad which did not touch the needle electrodes. The left and middle panels show the compound electrode without and with this pad. A schematic diagram of the compound electrodes is presented in the right panel.

Tactile and nociceptive electrocutaneous stimuli were applied using the compound electrode arrays we presented in an earlier paper (Steenbergen et al., 2012a); this electrode is presented in Figure 1. The devices consist of an array of disc and needle electrodes which are capable of eliciting a tactile or nociceptive sensation, the strength of which can be varied using pulse train modulation.

**Figure 2:**
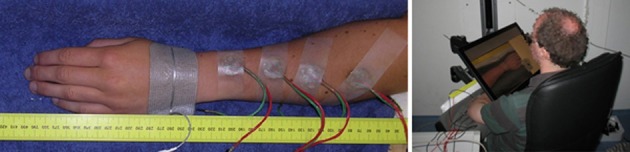
**Left:** The arm of a subject with four compound electrodes (green and red wires) and a reference electrode on the hand. **Right:** A subject seated using the tablet screen setup. The arm with electrodes is obscured from the subject's view by the monitor, which shows a photograph of the subject's own arm without electrodes.

Four compound electrodes were placed on the left lower arm along a line connecting the distal end of the ulna and proximal end of the radius. The most proximal electrode was placed 10 cm from the proximal end of the radius, the most distal one 4 cm from distal end of the ulna. The remaining two electrodes were placed with equal distance between all four electrodes. A Protens 9 × 5 cm rectangular TENS electrode served as anode and was placed at the wrist (see Figure 2). The electrodes were fixed using tape. The stimuli were applied using 8-channel stimulators with a common anode which were similar to stimulators in previous studies by our group (van der Heide et al., 2009; Roosink et al., 2011; Steenbergen et al., 2012a; van der Lubbe et al., 2012). All stimuli were monophasic cathodic pulses with a pulsewidth of 0.21 ms.

### Reporting method

Subjects performed the localization task on a 46 × 29 cm tablet monitor displaying a photograph of their own arm (see Figure 2). The monitor was placed over their arm, thus preventing visual information about the electrode positions from interfering with the experiment. When the electrodes were attached, subjects were prevented from seeing their arm by this same tablet monitor. The photograph was taken before the electrodes were placed, after which it was scaled in such a way that subjects reported that size and position matched the real arm. During the experiments, subjects reported the perceived stimulus locations by tapping the monitor using a pen, after which they tapped a ready button.

### Sensation thresholds

Sensation thresholds for the two electrode types were determined for each of the four sites using an adaptive psychophysical threshold determination method. The method consisted of applying a series (10 in this experiment) of stimuli of ascending amplitude. Based the estimated threshold and its uncertainty, the starting point and increment of the series was adjusted. The sensation threshold was defined as the current at which a subject has a 50% chance of detecting a stimulus. This point was determined using a logistic regression fit. Details of this method are presented in Steenbergen et al. (2012a). The sensation thresholds for the needle and disc electrodes were 0.62 ± 0.30 (M ± SD) mA and 2.76 ± 1.10 mA respectively.

### Stimuli

During the remainder of the experiments, the stimuli were pulse trains of five monophasic cathodic pulses, each of which had a pulse width of 0.21 and 5 ms between the onsets of the pulses. The amplitude of the stimuli was equal to the sensation threshold as determined for each site and electrode type combination.

### Qualitative verification of stimuli

We verified the quality of the eight stimuli which were to be used during the remainder of the experiment by using the quality visual analogue scale (VAS) which is described in Steenbergen et al. (2012a). The VAS was presented horizontally with left side being labeled as *dull* and the right side as *sharp*. The ratings were converted to numbers ranging from 0 (dull) to 10 (sharp). Reports lower than 5 were interpreted as tactile sensations, and higher than 5 as nociceptive. If the reported quality scores of an electrode were higher than 5 for the discs or lower than 5 for the needles, the electrode was moved to another spot on the skin. This was followed by a re-determination of the sensation thresholds and a new quality judgment. For the stimuli which were used in the localization experiments, subjects reported quality scores lower than five (dull halve of the scale) in 60 out of 68 stimulus sites.

### Experiment procedure

After giving informed consent, subjects were seated in a chair. They placed their left forearm in a comfortable armrest which was placed before them. A photograph was taken of the arm in the armrest. Next, their view of the arm was obscured by placing a tablet monitor, which was later used for reporting tasks during the experiments, between their head and arm. After this, the electrodes were placed as described above, following which the tablet monitor was lowered over the arm. This was followed by the sensation threshold and quality verification procedures. After this, the main experiment started. The localization experiment consisted of two blocks, a tactile and a nociceptive one, the order of which was randomly balanced over the subject population. 15 stimuli for each site were applied in each block, leading to a total of 120 stimuli. The localization procedure lasted approximately 30 min. At the end of the experiment, a photograph was taken of the subject's arm with electrodes and placed in the armrest.

### Data preparation of localization trials

The following procedures were performed in Matlab (version 7.13.0. Natick, Massachusetts: The MathWorks Inc., 2011).

Localization data: The reports which were generated by the tablet screen setup were in the form of x-y coordinates in pixels. This two-dimensional data was reduced to a single dimension by applying principal component analysis on the data of each subject separately and retaining the first principal component. After this, outliers were detected separately for each subject by site by modality condition and discarded. Outliers were defined as being 1.5 times the interquartile distance removed from the median.

Electrode locations: The photographs of the arms with electrodes were scaled to match the representation of the arm which was presented to the subjects during the experiments. In this scaled figure, the electrode locations were manually identified. These localizations were subsequently projected obliquely on the first principal component of the data.

As a final step, the data and electrode locations projected on the first principal component were normalized to the subjects' arm length by using information about the electrode placement in relation to the anatomy.

### Group level analysis: linear mixed model

The dataset with all trials was analyzed using the linear mixed model (LMM) in SPSS 18.0 using the default settings. LMM's have several advantages compared to a repeated measures ANOVA. The method accounts for inter subject differences, which, as discussed in the introduction, are considerable in the case of localization data. Also, the model allows the inclusion of correlated data points, therefore we could include all localization trials in the analysis, rather than the mean of each condition as would be the case in a repeated measures ANOVA. The model contained fixed main effects for *Stimulus type* (categorical with levels Tactile, for the disc electrode stimuli, and Nociceptive, for the needles electrode stimuli), *Electrode site* (a covariate ranging from 0 at the elbow to 1 at the wrist) and for the *Stimulus type* × *Site* interaction effect. A random intercept for subjects was modeled, as well as random effects for *Stimulus type* and *Electrode site*. A variance components covariance structure was used for modeling the random effects.

### Subject level analysis

In order to determine whether differences in localization are present at the subject level, we assessed each electrode site separately for each subject. For each site, a two-sided *t*-test was performed (using the Matlab *t*-test2 procedure) on the localizations of the tactile and nociceptive stimulus conditions. In addition, the magnitude of the difference in means was assessed. If this difference was larger than the maximum distance between disc and needle electrodes (1.5 cm), the difference was considered relevant. The reason for this cut-off value was that since each electrode array contains multiple electrodes of each type, there is a possibility that a single disc and a single needle are responsible for the stimuli because of differences in electrical contact between the component electrodes in each array. The maximum difference in stimulus site caused in this way in one compound electrode is 1.5 cm.

Performing separate *t*-tests for each site and subject means that 68 *t*-tests were performed for the whole dataset. In order to find out whether these differences were caused by false positives we counted the number of subjects which had one or more significant *t*-test result. This number was tested against the false positive rate using a binomial test. If we take the significance level for the *t*-tests of *p* = 0.05 as a worst case estimation for the probability of a false positive, the chance of any subject having one or more sites turn out positive by chance is 1–0.95^4^ = 0.186. The binomial distribution used for testing the number of subjects with significant *t*-tests was therefore B(17,0.186).

In addition to the tests of separate electrode sites, a separate regression model was fitted to the tactile and nociceptive localization datasets of each subject using the Matlab glmfit function. Trials were weighted such that each electrode site contributed equally to the regression fits. The intercepts and slopes of each were stored for analysis.

### Agreement between stimulus modalities

In order evaluate the agreement between perceptual maps of the tactile and nociceptive experiment conditions, we calculated intraclass correlation coefficients [*ICC(1,k)*, see (Shrout and Fleiss, 1979)] of slopes and offsets of the subject and modality dependent regression fits on the localization data. As a reference to compare these values with, we also calculated the within-modality reproducibility by splitting the data of each modality in two parts over time. This split was performed separately for each electrode in each subject to prevent unequal numbers of trials in the two halves. The difference between the split data ICCs and the between-modality ICCs was tested using Konishi-Gupta modified *Z*-tests (Donner and Zou, 2002).

## Results

**Figure 3:**
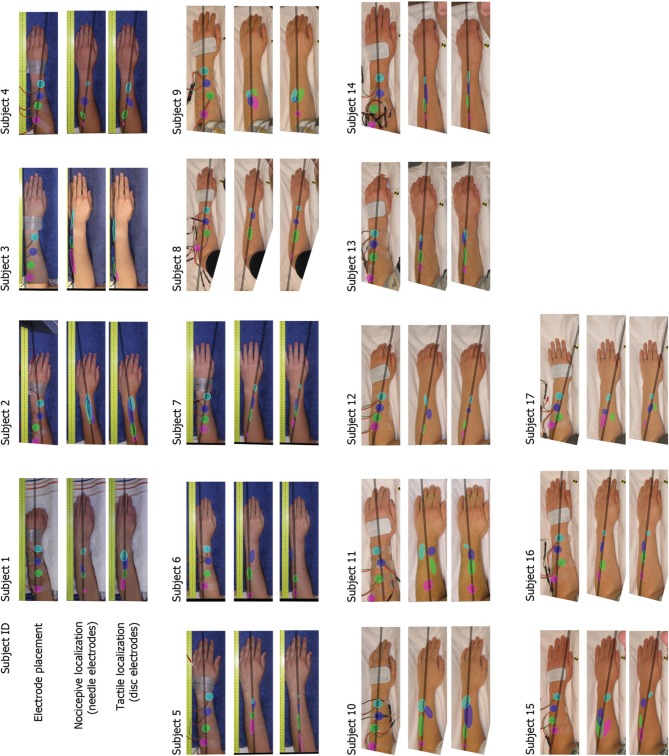
**Electrode placements and localizations plotted on the subjects' arms.** The localizations are plotted as means with standard deviations in two directions (the orientation of the ellipses was determined by applying a principal component analysis on all separate electrodes). The grey bar represents the first principal component of all data of each subject, this was the line on which all data is projected for further analysis.

**Figure 4:**
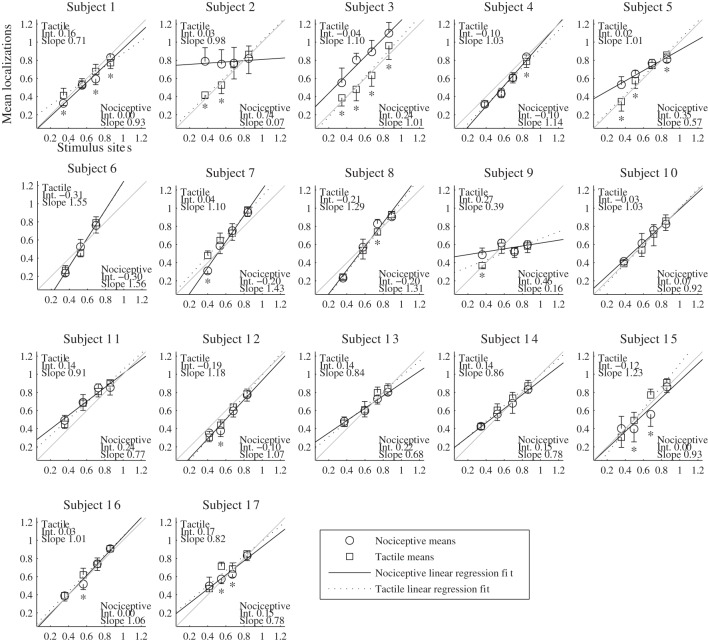
**Results of the localization experiments as a function of the stimulus site for each subject.** The localizations (vertical axis) are plotted as function of the sites (horizontal axis); both localizations and sites are shown as fraction of each subject's arm length, with 0 being the elbow and 1 the wrist. Each marker represents the mean localization of one site for either the nociceptive (circle) or the tactile (square) stimulus condition. The bristles indicate the standard deviations; these are plotted to one side only. The lines show the fitted regression models, solid for the nociceptive stimulus condition and dashed for the tactile condition. As a reference, a grey line is included in each panel which indicates the relation of perfect correspondence (i.e., intercept = 0 and slope = 1). The coefficients of the regression models are printed in the corners of each plot. Asterisks mark the sites for which (1) the localizations of the two types differed significantly and (2) the differences of the means were larger than the electrode diameter of 1.5 cm.

The results of the localization experiments projected on the subjects' own arms are presented in Figure 3. The top panels show the arms with electrodes, the middle and lowers panels the nociceptive and tactile localizations respectively. The localizations of each electrode are color coded to match the top panels. The localizations are drawn as means and SDs in two directions. The grey lines indicate the first principal component of the data of each subject to which the data was projected for further analysis. Figure 4 presents this reduced localization data as a function of the actual electrode positions, along with the linear regression fits for each stimulus type. These regression models showed the same features which were previously identified by Trojan et al. (2006): subjects showed contractions/expansions (slope larger/smaller than 1) and distal and proximal displacements (intercept smaller/larger than 1). In 12 out of 17 subjects at least one stimulus site showed a significant difference between localizations of tactile and nociceptive stimulus conditions which exceeded the electrode array diameter 1.5 cm (see Figure 4). Comparing this frequency of 12 out of 17 to a false positive rate 0.186 using a one-tailed binomial test showed that this number is significant (*p* < 0.001).

The agreement of the tactile and nociceptive regression parameters was *ICC(1,k)* = 0.66 [0.09–0.88 confidence interval (CI)] for the intercept and 0.76 [0.37–0.91 CI] for the slope. The ICCs for the slopes of the split data was 0.96 [0.90–0.99 CI] for the nociceptive and 0.98 [0.94–0.99 CI] for the tactile localizations. These ICCs were significantly higher than the between-modality ICC of the slopes (Konishy–Gupta modified *Z*-test: *T*_ZM_ = 3.67, df = 32, *p* < 0.001 and *T*_ZM_= 4.48, df = 32, *p* < 0.001 respectively). For the intercepts, the split data ICCs were 0.97 [0.91–0.99 CI] for the nociceptive and 0.96 [0.90–0.99 CI] for the tactile localizations, which in both cases was significantly higher than the between-modality ICC of the intercepts (*T*_ZM_ = 4.47, df = 32, *p* < 0.001 and *T*_ZM_= 4.41, df = 32, *p* < 0.001).

**Figure 5:**
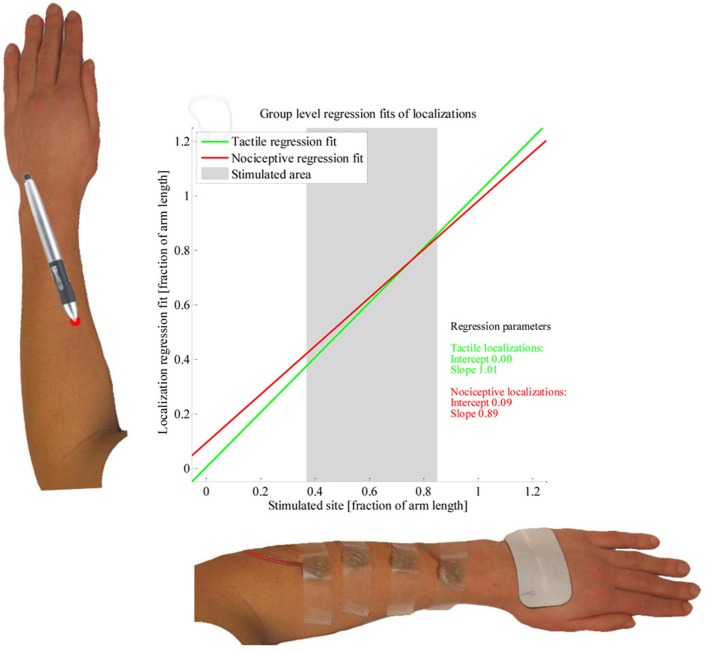
**Linear mixed model fit of the localization data.** The two lines present the group-level regression fits of the tactile (green) and nociceptive (red) localizations; the regression parameters are presented as well. Two example arms are presented along the axes. The average length over which subjects were stimulated is presented by a grey block.

The results of the LMM group level analysis are presented in Table 1. Significant effects were found for *Stimulus type*, *Electrode site* and for the interaction between these. The regression models fitted by the LMM on the tactile and nociceptive localizations of the whole study population are presented in Figure 5. Both the stimulus sites and localizations are represented as a fraction of the arm length, with 0 being at the elbow and 1 at the wrist.

**Table 1:** **Linear mixed model fixed effects results for the normalized localizations**.

**Factor**	**df^a^**	***F***	***p***
Stimulus type	1/40.1	16.00	<0.001^*^
Electrode site	1/15.8	178.06	<0.001^*^
Type × Site	1/1825.0	30.79	<0.001^*^

* *p < 0.05*,

a Numerator/denominator degrees of freedom.

## Discussion

Our aim was to determine whether there are differences between tactile and nociceptive perceptual maps at the subject level and whether the perceptual maps of these modalities are in agreement for the whole subject population. In a series of experiments, tactile and nociceptive sensations were elicited on the lower arm using electrocutaneous stimulation. Subjects repeatedly reported the perceived location of stimuli of both types applied at four sites. When assessed for each separate electrode, the localizations of the tactile and nociceptive conditions differed for most subjects, but in a manner which varied between subjects. Despite these differences, linear regression fits of the data of each separate modality and subject showed some measure of agreement. This agreement was lower than the within modality ICCs we obtained by splitting the data in two halves.

The subject-level differences that we found between tactile and nociceptive localization occurred at different sites and do not seem to follow a common pattern for all subjects. In some subjects, no difference in localization between the two modalities was found. Nevertheless, from the magnitude of the differences and their frequent occurrence we conclude that these differences in localization are the result of an actual difference in perceived location and not a chance occurrence. From our data we cannot conclude what causes these differences. Possibly they reflect the columnar organization of the primary sensory cortices for touch and nociception.

At the group level, we found the regression fits of the tactile and nociceptive stimuli to differ, with the regression fit of the nociceptive localizations being contracted while the fit of the tactile localizations was close to veridical. This matches the results by Mancini et al. (2011), who found group-level perceptual maps of painful laser stimuli on the dorsal and volar hand to be more contracted than perceptual maps of tactile stimuli. However, since the regression fits for individual subjects show large differences, performing the same experiment in a new population is likely to yield different group level results.

The moderate agreement we found between tactile and nociceptive perceptual maps is consistent with a common body representation underlying perception of these modalities, which supports the conclusions by Mancini et al. (2011). However, this agreement was less than the agreement within each modality as calculated by splitting the data of each subject in two over time. Also, there were significant differences between modalities in most subjects. This indicates that common body representations and the physical location of the stimuli together do not fully account for the differences in perceptual maps between modalities. Another factor is responsible for these differences, possibly a difference in primary representations in the somatosensory cortex. Thus our findings are consistent with a projection of information from slightly different primary representations to common body representations. Differences in perception between touch and nociception are unlikely to be noticeable in daily life. Multisensory integration processes have been demonstrated to be able to integrate spatially disparate information from different modalities into a single percept (Alais and Burr, 2004; Block and Bastian, 2009). In any real life situation, information from various cutaneous senses generally arises in conjunction, therefore a difference in spatial perception between touch and nociception due to a difference in primary representations would not be noticeable due to these integration processes. Making a difference in spatial perception between touch and nociception observable requires eliminating or minimizing the integration of nociceptive information with tactile cues, for which we used electrocutaneous stimulation.

Although we found significant differences in tactile and nociceptive localization, other stimulus parameters than modality could have contributed to these. Very little information is available about the effect of stimulus intensity (both physical and perceived) and stimulus duration on localization. Hamburger (1980) reported an increase in localization accuracy with increasing force of mechanical stimulation, but it is unknown whether this difference is due to a reduction in the stochastic component of localizations or to effects on the perceptual map. Since touch and nociception are different modalities, perceived strength of these cannot be directly compared. Concerning physical stimulus strength, we already demonstrated in the previous publication that varying the physical strength of electric stimuli using pulse train modulation has a different effect on perceived intensity for preferential stimulation of touch and nociception (Steenbergen et al., 2012a). Therefore a possible effect of stimulus intensity on localization is likely to differ between these modalities.

In conclusion, we found perceptual maps of electrically elicited nociceptive and tactile stimuli to differ. We suggest that differences in primary representations between the modalities may be responsible for these differences. We also found moderate agreement between perceptual maps of both modalities, which is consistent with the involvement of common underlying internal body representations. Further research will have to point out whether the differences we found are indeed due purely to a difference in stimulus modality, or whether another stimulus parameter contributed to this.

### Conflict of interest statement

The authors declare that the research was conducted in the absence of any commercial or financial relationships that could be construed as a potential conflict of interest.
